# An In-Vitro Optical Sensor Designed to Estimate Glycated Hemoglobin Levels

**DOI:** 10.3390/s18041084

**Published:** 2018-04-04

**Authors:** Sanghamitra Mandal, M. O. Manasreh

**Affiliations:** Department of Electrical Engineering, University of Arkansas, Fayetteville, AR 72701, USA; manasreh@uark.edu

**Keywords:** optical sensor, glycated hemoglobin (HbA1c), absorbance spectroscopy, Beer’s law, diabetes

## Abstract

The purpose of this research was to design an optical sensor for evaluating glycated hemoglobin (HbA1c) percentages in hemoglobin. The A1c sensors available in the market use invasive methods, while our device offers the possibility of non-invasive monitoring of HbA1c levels in diabetic patients. A prototype is assembled using two light emitting diodes with peak emission wavelengths of 535 nm and 593 nm, a photodiode, and a microcontroller. The proposed sensor measures the transmitted intensity in the form of an output voltage. We devise an approach to estimate the percentage of HbA1c in hemoglobin for a given solution. This estimation is based on the relative change in absorbance due to change in path length and molar absorption coefficients of hemoglobin and HbA1c, at the two wavelengths. We calculate the molar absorption coefficient of HbA1c at 535 nm and 593 nm wavelengths using the sensor, which is performed by a multiple variable regression analysis algorithm fed through the microcontroller. Specifically, the sensor output voltage with respect to the sample concentration is fitted to an exponentially decaying equation model. We used a commercial chemical assay called Control FD Glycohemoglobin A1c with known percentage HbA1c levels to verify our device measurements.

## 1. Introduction

Diabetes mellitus is a serious metabolic disease that severely affects over 422 million people around the world [1]. Diabetic patients are twice as likely to be affected by heart diseases, kidney failure, stroke, eye cataracts, feet amputation, or sudden mortality. Therefore, frequent glucose monitoring is vital for adjusting treatment and maintaining normal blood glucose levels. Over the past four decades, enzymatic and non-enzymatic electrochemical glucose sensors have emerged as the most investigated device technologies in this area [2,3,4,5,6,7]. The glucose monitoring sensors available on the market are mostly electrochemical sensors that are both economical and highly accurate [3,8], but require pricking of finger tissues several times a day to extract capillary blood. The long-term disadvantages of using such invasive needle-based glucose sensors are damaged finger tissues, excessive pain experience, and high risks of infections like tetanus. Other disadvantages of invasive sensors include inaccurate sensor measurements due to noise and patients’ movements, and skin irritations caused by direct sensor contact with dermal tissue.

In the past, several types of sensors for glucose sensing have been investigated and developed. However, non-invasive glucose sensor technology is the most recent technique that is pain free and refers to direct measurement of glucose levels through body tissues (skin, eyes, or the tongue/saliva) [9]. The review by Bruen et al. [10] focuses on various non-invasive glucose monitoring approaches using biological fluids like interstitial blood, sweat, breath, saliva, and ocular fluid. Lately, various alternative approaches have being explored to develop cost effective and highly sensitive glucose sensors for precise glycemic control, like reverse iontophoresis [11], tear glucose dynamics [12,13], and dielectric spectroscopy [14]. Conceptually, optical sensors are the least invasive form of biological instruments, yet testing glucose non-invasively using optical methods has not yielded consistent results so far according to the literature [15]. Since the 1980s, HbA1c concentration started to be accepted as a clinical standard to assess the blood glucose levels in diabetic, pre-diabetic, and pregnant diabetic patients. The challenges involved in using HbA1c as a reliable tool in the routine testing and management of glycemic state were very well described by Weykamp [16]. 

The estimation of long-term glycemic level in blood is performed using a compound called glycated hemoglobin (HbA1c) found in red blood cells. Hemoglobin (Hb) A1c is a minor red cell constituent that comprises 5% of the total Hb in normal individuals but up to 15% in patients with poorly controlled diabetes mellitus [17]. The HbA1c level is tested to indicate the average blood glucose level over the past 8–12 weeks [18] since the average functional lifespan of red blood cells in human body is about 120 days. In 1976, HbA1c was first used to monitor the degree of control of glucose metabolism in diabetic patients [19]. Since then several studies have been conducted to standardize HbA1c level in correlation to the average glucose measurements [20]. The American Diabetes Association has established Equations (1) and (2) to calculate the estimated Average Glucose (eAG) level from the percentage of HbA1c in blood hemoglobin [20,21]:

(1) eAG (mmol/L)=1.59 ×HbA1c (%) −2.59

(2) eAG (mg/dL)=28.7 ×HbA1c (%) −46.7

In this paper, we report the possibility of designing an optical HbA1c sensor. We begin with the description of the experimental setup and then define the chemical assays used to conduct the experiments. Next, we discuss the molar absorbance spectrum, and the calculation of molar absorbance coefficient of the samples using the setup. Calculations are executed by means of multiple variable regression analysis. Finally, we describe the steps used to estimate the percentage of HbA1c in the glycohemoglobin A1c samples.

## 2. Methodology

A portable in vitro sensor is designed for the estimation of HbA1c percentage level. The major device components include two commercially purchased light emitting diodes (LEDs), a cuvette holder, a photodiode, and an ATmega328 microcontroller. The two LEDs used in the setup are: (1) green LED (HLMP-CM1A-560DD, Broadcom Limited, San Jose, CA, USA) with a peak spectral emission of 535 nm and a bandwidth of 33 nm and (2) yellow LED (TLCY5800, Vishay Semiconductor, Hicksville, NY, USA) with a peak spectral emission of 593 nm and a bandwidth of 11 nm. Disposable polystyrene cuvettes with path length of 1 cm are used to hold the chemical assays during the test. A silicon photodiode (FD11A, Thorlabs, Newton, NJ, USA) is used as the light detector that detects the photons emitted by the LED and transmitted through the chemical assays. The photodiode detection ranges from a wavelength of 320–1100 nm of the visible spectrum. The Uno R3 is an 8-bit microcontroller (Arduino, made in Italy) built onto a single printed circuit board based on the ATmega328P (AVR microcontroller Atmel, San Jose, CA, USA) with a 32 KB of flash memory and 2 KB of random access memory. Appendix A presents the code written in C programming language. This code is used for the measurement of photodiode voltage response, and the calculation of molar absorption coefficient via multiple variable regression. 

**Figure 1:**
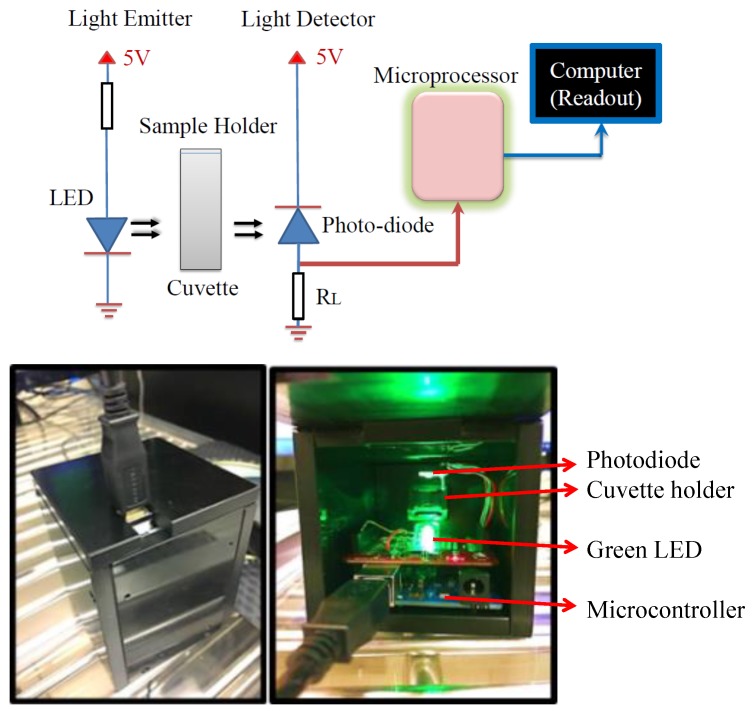
(**a**) The schematic of the experimental setup used to calibrate and test glycated hemoglobin concentration. (**b**) Snapshots of the designed prototype.

Figure 1 represents the schematic of the experimental setup. The room temperature electroluminescence of the commercial green and yellow LEDs is measured using the LabRAM HR Evolution spectroscope (Horiba, Irvine, CA, USA) while biased through a 2410 source meter (Keithley, Cleveland, OH, USA). A Cary 500 Scan UV-Vis-NIR spectrophotometer (Varian, Palo Alto, CA, USA) is used to measure the absorbance of the reagents used in the experiment. The current prototype is tested by means of a commercially purchased chemical marker called Control FD Glycohemoglobin A1c Level-2 (Product No. K061M-6, Lot No. 06621, Audit MicroControls, Eatonton, GA, USA) [22]. It is a reference control consisting of human blood-based solutions intended to simulate human blood samples containing HbA1c. Various laboratories utilizing FDA approved instruments and reagents have estimated the percentage of HbA1c in the chemical marker Control FD Glycohemoglobin A1c, and reported it to be in the range from 8–13% with a mean around 10% [22]. The pH for glycohemoglobin A1c is measured using a digital pH meter (PHH-7011, Omega, Stamford, CT, USA) and is observed to be equal to 6.85 at 19.5 ℃. We also measure the absorbance for another chemical marker called Hemotrol (HemoTrol-Level 3-Lot 63667, Eurotrol, Elizabethtown, KY, USA), which is formulated with real hemoglobin to closely mimic whole blood [23]. We use crystal violet dye (32675, (Sigma Aldrich, St. Louis, MO, USA) diluted in deionized water, and rhodamine 6g dye (R4127, Sigma Aldrich) dissolved in ethyl alcohol, to validate the molar absorption coefficients estimated using our device against the previously reported results. 

## 3. Results and Discussion

Here we study the feasibility of designing an optical HbA1c sensor to be able to indicate average glucose level in the last 100–120 days. The working principle of the proposed optical sensor is based on Beer’s law of optical absorption. According to Beer’s law, the absorbance (A) of the light wave of wavelength, λ with an intensity (I_0_) passing through a solution of concentration, x (mol L^−1^) over the path length, l is given by Equation (3) [24,25]. In other words, absorbance (A) is defined as log of ratio of transmitted intensity (I) to incident intensity (I_0_) of the light wave Equation (3). The proportionality constant, ε (L mol^−1^cm^−1^) is called the molar absorptivity or the molar absorption coefficient for a given sample that depends upon the specified wavelength (λ). The transmitted intensity of light (I) as a function of wavelength (λ) is given by Equation (4):

(3) $$A\left(\lambda \right)=-\ln\left(\frac{I}{I_{0}}\right)=\varepsilon \left(\lambda \right)\times l\times x$$

(4) $$I\left(\lambda \right)=I_{0\;}*\exp(-\varepsilon \left(\lambda \right)\times l\times x)$$

The properties of the Beer’s law are valid even if more than one material absorbs light in the medium. Each absorber contributes to the total absorbance and resulting total absorbance A_total_ is a superposition of the individual absorbing processes. The total absorbance of hemoglobin due to glycated and non-glycated hemoglobin is given by Equation (5): 

(5) A_total_ = A_Hb,A1c_ + A_Hb,NonA1c_ = ε_Hb,A1c_ × 1 × x_Hb,A1c_ + ε_Hb,NonA1c_ × 1 × x_Hb,NonA1c_

Here we consider that all the parameters are averaged over oxyhemoglobin and deoxyhemoglobin. The arterial blood in normal humans comprises of roughly 98% oxy-hemoglobin and 2% deoxyhemoglobin [26]. The percentage of HbA1c (% HbA1c) in total hemoglobin in terms of molar concentrations of HbA1c (x_Hb,A1c_), non-glycated hemoglobin (x_Hb,NonA1c_) and total hemoglobin (x_Hb_) is given by Equation (6):

(6) $$\%\;\mathrm{HbA}1c=\frac{x_{\mathrm{Hb},\;A1c}}{x_{\mathrm{Hb},A1c}+x_{\mathrm{Hb},\;\mathrm{Non}\;A1c}}\times 100=\frac{x_{\mathrm{Hb},A1c}}{x_{\mathrm{Hb}}}\times 100$$

We calculated the % HbA1c in total hemoglobin using parameter R and the molar absorption coefficient for glycated hemoglobin (ε_HbA1c_) and non-glycated hemoglobin (ε_HbNonA1c_) using Equation (7). The parameter R is defined as the ratio of change in absorbance as the path length changes (l_1_ to l_2_) at two different wavelengths λ1 and λ2 (Equation (8)). The detailed derivation of Equations (7) and (8) are stated in Appendix B. 

(7) $$\%\;\mathrm{HbA}1c=\;\frac{\varepsilon_{\mathrm{Hb},\mathrm{NonA}1c}\left(\lambda 1\right)-R\times \varepsilon_{\mathrm{Hb},\mathrm{NonA}1c}\left(\lambda 2\right)\times 100}{R\times \left(\varepsilon_{\mathrm{Hb},\mathrm{NonA}1c}\left(\lambda 2\right)\;-{\;\varepsilon }_{\mathrm{HbA}1c}\left(\lambda 2\right)\right)-\left(\varepsilon_{\mathrm{HbNonA}1}\left(\lambda 1\right)-\varepsilon_{\mathrm{HbA}1c}\left(\lambda 1\right)\right)}$$

(8) $$R=\;\frac{{\delta A}_{\lambda 1}}{{\delta A}_{\lambda 2}}=\frac{\ln{\frac{I\left(l1\right)}{I\left(l2\right)}}_{\lambda 1}}{\ln{\frac{I\left(l1\right)}{I\left(l2\right)}}_{\lambda 2}}$$

The detailed derivation of Equations (7) and (8) is provided in Appendix B. The two wavelengths selected for the above calculations are based on the selective absorbance of HbA1c in the wavelength ranging from 520–610 nm (discussed in Section 3). Similar principle is employed in pulse oximetry or photoplethysmography to recognize oxygen saturation only for the arterial compartment of blood [25]. 

The molar absorbance coefficients used in Equation (7) for ε_HbNonA1c_ is derived from [27] and ε_HbA1c_ is estimated using our device. More specifically we use multi-variable regression analysis method described as follows. The transmitted intensity of the LED after it passes through the sample for a fixed path length (l), varies with respect to the sample concentration (x) [Equation (4)]. The proposed sensor measures the transmitted intensity in the form of photodiode output voltage. The output voltage is then plotted as a function of concentration that is then fitted along an exponentially decaying expression Equation (9):

(9) y=A+B×exp(−C×x)

In Equation (9) A, B, and C are three unknown variables, x is the concentration, and y is the output voltage. Equation (9) is modelled against the transmitted intensity Equation (4). By comparing Equations (4) and (9), we get molar absorption coefficient ε = C/l, where l is the path length of the cuvette, equal to 10 mm.

**Figure 2:**
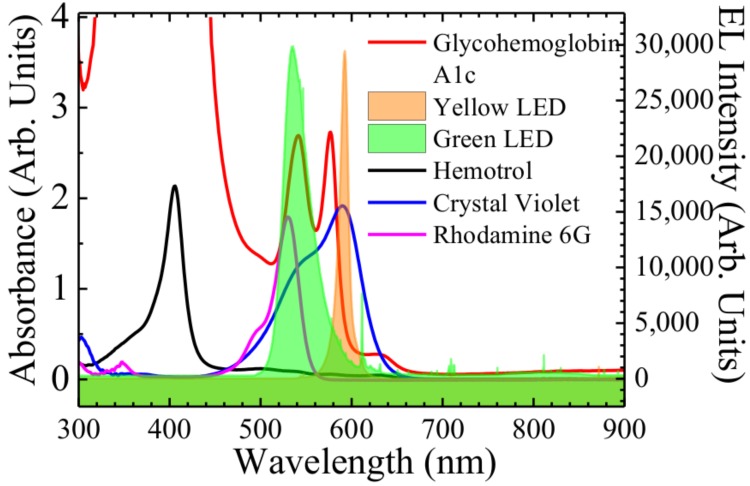
Electroluminescence of green and yellow LED measured at room temperature compared to the absorbance of Glycohemoglobin A1c, Hemotrol, crystal violet, and rhodamine 6 g solutions.

The first objective of our analysis is to determine the wavelengths at which we observe selective absorbance due to glycated hemoglobin (HbA1c) in blood hemoglobin. Figure 2 represents the absorption spectra of Control FD Glycohemoglobin A1c and Hemotrol. It is observed that glycohemoglobin A1c demonstrates strong absorption around 412 nm (Soret Band), and 541 nm and 577 nm (Q Bands). However, Hemotrol has a very strong absorption only around 405 nm (Soret Band) and weak absorption around 499 nm, 576 nm, and 630 nm (Q Bands) wavelength range. Since, glycohemoglobin A1c shows a strong absorbance in the wavelength range of 520–610 nm and Hemotrol does not, we use green and yellow LEDs as the light emitter in the proposed setup. In Figure 2 we also show the electroluminescence (EL) intensities of the green and yellow LEDs. It is seen that the peak emission wavelength for green LED is at 535 nm and the full width at half maximum spectral bandwidth is about 33 nm. The yellow LED has a peak emission spectrum at 593 nm and the full width at half maximum spectral bandwidth is about 11 nm. The overlapping of the EL emission spectra of the green and yellow LEDs, and the absorbance spectrum of HbA1c show that the green LED used in the setup can very well be used to detect HbA1c. We also measure the absorbance of two dyes to verify our method of estimating molar absorption coefficient. It is understood from Figure 2 that the absorbance spectra of rhodamine 6 g, and crystal violet overlaps the EL spectra of the green, and yellow LED, respectively.

### 3.1. Molar Absorption Coefficient Calculation

**Figure 3:**
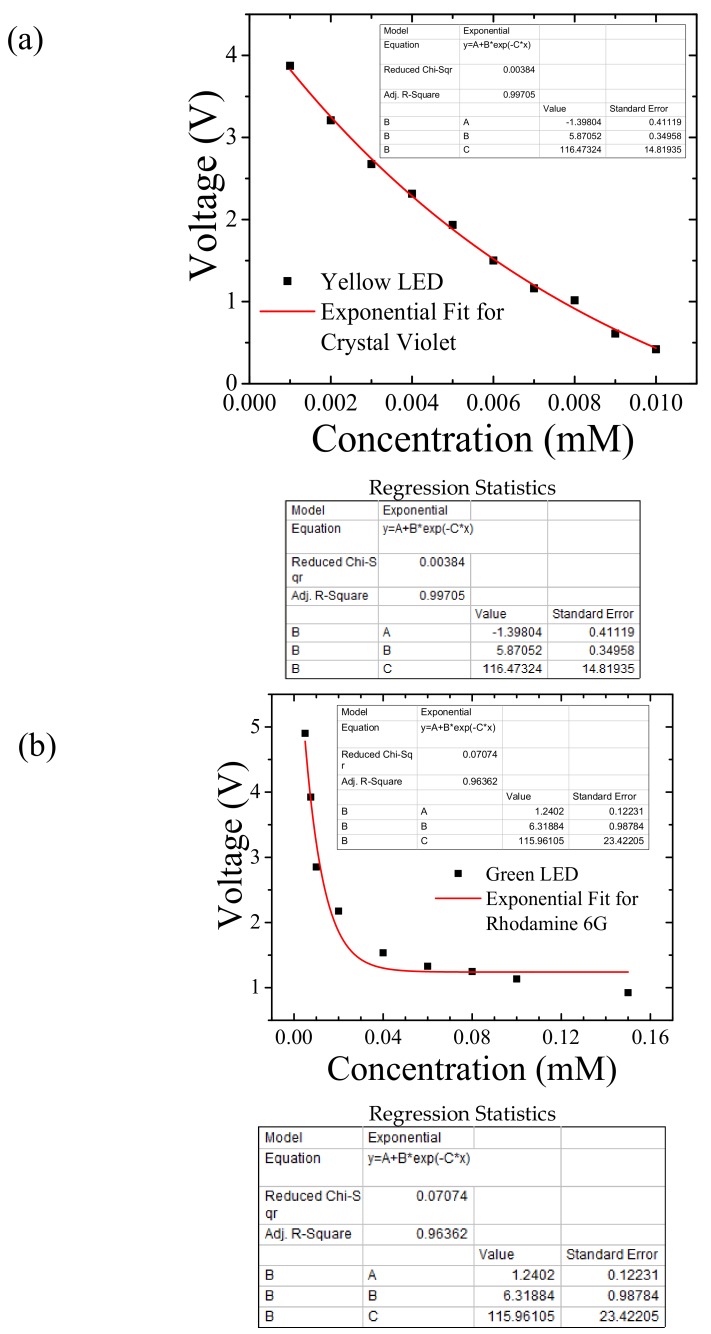
Photodiode output voltage (averaged over 500 voltage readings) as a function of concentration of (**a**) Crystal Violet synthetic dye using the yellow LED and (**b**) rhodamine 6 g fluorescent dye using the green LED. The data points are fitted to an exponentially decaying equation model via a three-variable regression analysis to find molar absorption coefficient.

We show the photodiode voltage response with respect to the concentrations of crystal violet dye using the yellow LED in Figure 3a and rhodamine 6 g dye using green LED in Figure 3b. In Figure 3a,b each of the voltage data points corresponding to the sample concentrations are averaged over 500 voltage readings. The regression statistics of Figure 3a show an adjusted R-square value of 99.71%, which indicates how well the data points fit the exponential model. In Figure 3b, the adjusted R-square value is 96.36%, which indicates the goodness-of-fit for our regression model. The voltage data points then are fitted (red line) to the exponentially decaying function model given by Equation (9). From the value of variable *C*, the molar absorption coefficient for crystal violet at yellow light wavelength is 116,478.24 ± 14,819.35 M^−1^cm^−1^ and for rhodamine 6 g at green light wavelength is 115,961.05 ± 23,422.05 M^−1^cm^−1^. These calculated molar absorption coefficients for crystal violet and rhodamine 6 g are in good agreement with the published values of crystal violet, 112,000 cm^−1^M^−1^ [28] and rhodamine 6 g, 116,000 cm^−1^M^−1^ [29]. Thus, the proposed method reliably estimates the molar absorption coefficient for a given solution.

**Figure 4:**
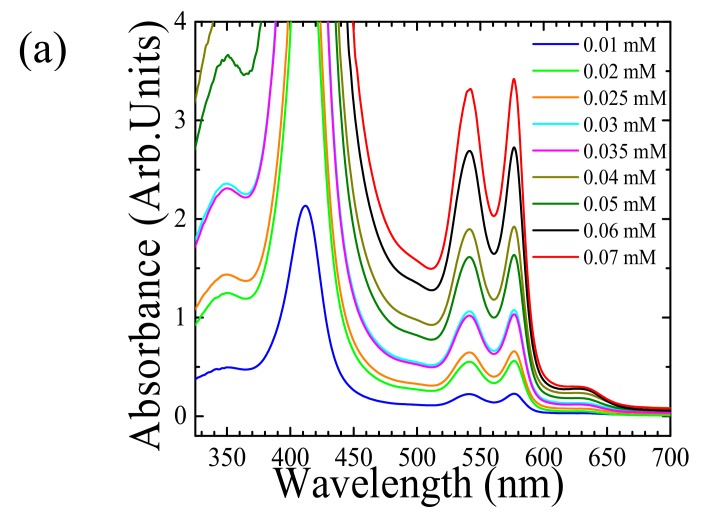

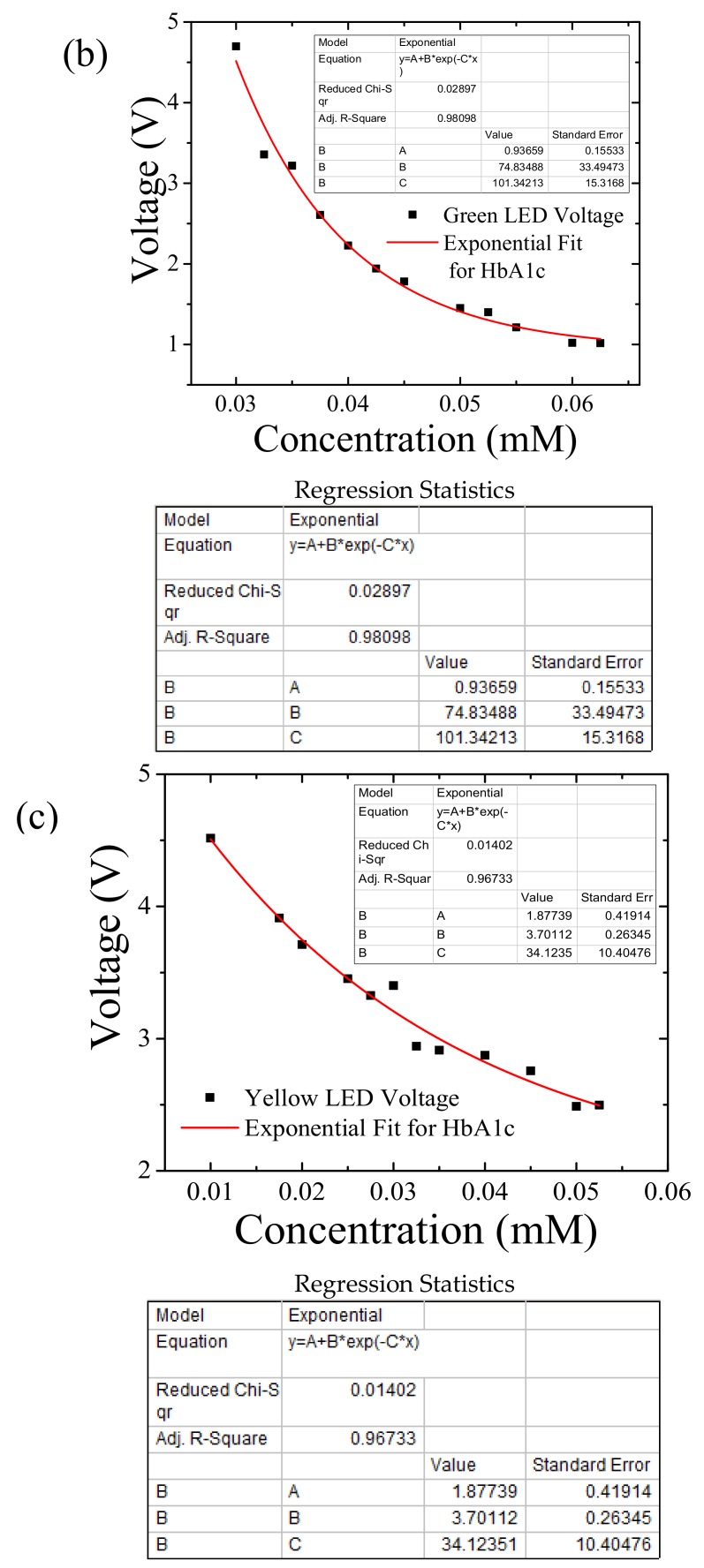
(**a**) The absorption spectra of diluted concentrations of glycohemoglobin A1c solutions measured at room-temperature. (**b**) Photodiode output voltage (averaged over 500 voltage readings) as a function of concentration for diluted concentrations of glycated hemoglobin using green LED and (**c**) yellow LED. The data points are fitted to an exponentially decaying equation using a three-variable regression analysis.

Figure 4a presents the room temperature absorbance spectra of glycohemoglobin A1c solutions for concentrations ranging from 0.01–0.07 mM. It is observed that an increase in the concentration leads to an increase in the absorbance, specifically around 541 nm and 577 nm. This implies that as the concentrations increase, the transmitted light intensity of the green and yellow LEDs should decrease. The photodiode voltage response corresponding to different glycohemoglobin A1c concentration is shown in Figure 4b using green LED, and Figure 4c using yellow LED. Each of the voltage data points are averaged over 500 readings measured at the corresponding concentrations. The exponential fit variable C gives the molar absorption coefficient of glycohemoglobin A1c solution under green light to be, 101,342.13 ± 15,316.8 M^−1^cm^−1^. Similarly, the molar absorptivity of glycohemoglobin A1c solution under yellow LED is calculated as 34,123.5 ± 10,404.76 M^−1^cm^−1^. The marker Control FD glycohemoglobin A1c, comprises of glycated hemoglobin and non-glycated hemoglobin. Therefore, to calculate the molar absorption coefficient for 100% glycated hemoglobin, we factor out the absorbance due to non-glycated hemoglobin according to Equation (10). The detailed derivation of Equation (10) is specified in Appendix C:

(10) $$\varepsilon_{\mathrm{Hb},A1c}=\frac{100\times {\;\varepsilon }_{\mathrm{Hb}}-{\;\varepsilon }_{\mathrm{Hb},\mathrm{Non}\;A1c}\times \;\left(100-\%\mathrm{Hb},A1c\right)}{\%\mathrm{HbA}1c\;}$$

The molar absorption coefficient of non-glycated hemoglobin${\;\varepsilon }_{\mathrm{Hb},\mathrm{Non}\;A1c}$ is 48,338 M^−1^cm^−1^ at 535 nm wavelength (green LED) and 9073.6 M^−1^cm^−1^ at 593 nm wavelength (yellow LED) [27]. The estimated molar absorption coefficient of the marker glycohemoglobin A1c solution is represented by $\varepsilon_{\mathrm{Hb}}$. Since the percentage of HbA1c level of marker lies between 8–13% [22], we estimate the molar absorption coefficient of HbA1c,${\;\varepsilon }_{\mathrm{Hb},A1c}$ value for both the upper and lower limits. Using Equation (10),${\;\varepsilon }_{\mathrm{Hb},A1c}$ is calculated to be 456,061 ± 117,823 M^−1^cm^−1^ (for, % HbA1c = 13%) and 710,888 ± 191,458 M^−1^cm^−1^ (for, %HbA1c = 8%), at 535 nm wavelength (green LED). Similarly, ${\;\varepsilon }_{\mathrm{Hb},A1c}$ is calculated to be 201,765 ± 80,037 M^−1^cm^−1^ (for, %HbA1c = 13%) and 32,2197 ± 130,060 M^−1^cm^−1^ (for, % HbA1c = 8%) at 593 nm wavelength (yellow LED).

### 3.2. Estimation of HbA1c Percentage in a Solution

Here, we estimate the molar absorption coefficient of glycohemoglobin A1c solutions by employing our device. However, one can estimate the molar absorption coefficients of the samples under test via other methods [30,31,32]. In that case the proposed sensor will only measure the value of R parameter using Equation (8). Next the percentage of HbA1c level is estimated through Equation (7) by substituting the pre-determined molar absorption coefficient values and the calculated R values. The calculation of R parameter is performed by measuring transmitted intensity for the samples at λ1 and λ2 equal to 535 nm (green LED) and 593 nm (yellow LED), respectively for the path lengths of 10 mm and 5 mm:

(11) $$R=\;\frac{\ln{\frac{I\left(10\;\mathrm{mm}\right)}{I\left(5\;\mathrm{mm}\right)}}_{\mathrm{Green}}}{\ln{\frac{I\left(10\;\mathrm{mm}\right)}{I\left(5\;\mathrm{mm}\right)}}_{\mathrm{Yellow}}}$$

Table 1 presents the observed R values for different samples of marker glycohemoglobin A1c calculated using the proposed sensor. Here, the molar absorption coefficients values of ${\;\varepsilon }_{\mathrm{Hb},\mathrm{Non}\;A1c}$ and ${\;\varepsilon }_{\mathrm{Hb}},$ declared in Section 3.1 are used for the percentage of HbA1c estimation [Equation (10)]. Table 1 lists the final percentage values of HbA1c calculated in the marker glycohemoglobin A1c solutions and are found to be in close agreement with the measurements from other laboratories [22].

## 4. Conclusions 

The use of glycated hemoglobin (HbA1c) sensors for tracking long term glycemic status in diabetic patients is more convenient as compared to the temporal information gained from blood glucose sensors. The currently available electrochemical HbA1c sensors are based on liquid chromatography, immunoassay, electrophoresis, or spectrophotometry techniques that require invasive blood extraction. There is a pressing need for the development of advanced non-invasive optical HbA1c sensors with better selectivity, stability, and accuracy. This research proposes the design of an inexpensive optical sensor prototype to calculate: (1) the molar absorption coefficients of a solution using multiple variable regression analysis, and (2) the percentage of HbA1c in a solution. The working principle of the proposed sensor is based on the Beer’s Law of optical absorption. The molar absorption coefficients measured for two known dyes using our prototype is in good agreement with the previously established results. Also, the calculated percentage of HbA1c in the solution falls within the standard range of values established by other laboratories. However it is important to mention that not all instruments measure values in good agreement with each other. A few of the possible sources of error include gravimetric and volumetric errors, path length errors, beam alignment errors, reflection errors, and errors due to stray radiation [33]. We use a range of molar concentrations of the chemical assay at which our current setup is the most sensitive. This is one of our device limitations. However, the concentration of whole blood is much higher and requires a setup enhancement by using a high sensitivity photodiode sensor and brighter LEDs. Further research and enhancement of our current prototype by testing pure glycated blood samples and actual human blood samples is required, which could eventually lead to a commercialized portable HbA1c sensor.

## Figures and Tables

**Table 1:** Estimated percentage of glycated hemoglobin.

Sample No.	Molar Concentration of Control FD HbA1c (mmol/L)	R	HbA1c (8%)	HbA1c (13%)
1	0.03	2.8206	10.31%	16.75%
2	0.035	2.9710	7.99%	12.98%
3	0.04	2.8641	9.54%	15.50%
4	0.05	2.9573	8.16%	13.27%
5	0.0525	2.9682	8.02%	13.03%

## Appendix A

#include <EEPROM.h>
#include <Math.h>
                                       // Sensor input variables
int sensorPin = A0;                   // select the input pin for the photodiode
int ledPin2 = 7;                       // select the pin for the LED
int sensorValue = 0;                  // variable to store the value coming from the sensor
int eeAddress = 0;                   // Location we want the data to be put in EPROM.
             //sequentially stores 10 readings from your analog sensor into an array, one by one.
             //With each new value, the sum of all the numbers is generated and divided
                                        //producing an average value
const int numReadings = 500;          // Number of sensor readings you want to take at one concentration                       
int total = 0;                           // sensor reading:
double volts = 0;
double current = 0;
double totalV = 0;                          // the running total Voltage
double totalI = 0;                          // the running total Current
double averageV = 0;                     // the average Voltage
double averageI = 0;                     // the average Current
double concentration = 0;               // sample concentration
int maxcount = 11;                     // No. of sample concentrations
//variables for exponential regression
double x[11];                  // always set the size of array equal to maxcount
double y[11];                 // always set the size of array equal to maxcount
double dx[10];               // always set the size of array equal to (maxcount-1)
double dy[10];              // always set the size of array equal to (maxcount-1)
double cx[10];                 // always set the size of array equal to (maxcount-1)
double dq[10];                // always set the size of array equal to (maxcount-1)
double b_initial[9];           // always set the size of array equal to (maxcount-2)
double a_initial[10];         // always set the size of array equal to (maxcount-1)
double b_int_avg=0;
double a_avg=0;
double c_initial[11];         // always set the size of array equal to maxcount
double lnyc[11];            // always set the size of array equal to maxcount
double xbar=0;
double ybar=0;
double xybar=0;
double xsqbar=0;
double slope=0;
double intercept=0;
double syx = 0;
double std_err = 0;
double stderr_Slope = 0;
double stderr_Intercept = 0;
double a=0;
double b=0;
double c=0;
double a_err=0;
double b_err=0;
double c_err=0;
int abor(double y[11],double cc)                   // always set the size of array equal to maxcount
{
  int returnpara=0;
  double e=0;
   for (int i=0; i<maxcount-1; i++)
       {
        e = (y[i+1]-cc)/(y[i]-cc);
        if(e<=0)
          {
           returnpara=1;
           break;
          }
         } 
  return returnpara;
}
                                   // always set the size of array y and x equal to maxcount, dx equal to maxcount-1
double Devi(double cc, double y[11], double x[11],double dx[10])  
{
   double avG=0;
   double pre_ee=0;
   double ee=0;
   double growthfactor[maxcount-1];
   double Deviation = 0;
   for (int i=0; i<maxcount-1; i++)
         {
          pre_ee = (y[i+1]-cc)/(y[i]-cc);
          ee = log(pre_ee);//denominator                   
          avG = avG + ee;
          growthfactor[i] = exp(ee/dx[i]);                    //save
         }
  avG = exp(avG/(x[maxcount-1]-x[0]));                   //exp added
  ee = 0;
  for (int i=0; i<maxcount-1; i++)
       {
       Deviation = Deviation + abs(avG-growthfactor[i]);
       }  
return Deviation;
}  
   void setup() 
        {                                           // initialize serial communication with computer:
         Serial.begin(9600);                        // Set data rate to 9600 bps
         pinMode(ledPin2, OUTPUT);
        }   
   void loop() 
   {
   digitalWrite(ledPin2, HIGH);  
   delay (3000);                                   // delay to start recording values or LED stabilizing delay 
   for (int j=0; j<maxcount; j++)
        {
         totalV = 0;
         total = 0;
         totalI = 0;
         for (int i = 0; i<numReadings; i++)
             {
               sensorValue = analogRead(sensorPin);           // read the value from the sensor:
               volts = double (sensorValue) * (5.0 / 1023.0);     // map it to the range of the analog out:
			   
               Serial.print(“\t Sensor = ”);                 // print the results to the serial monitor:
               Serial.println(sensorValue);
               Serial.print(“ Volts = ”);
               Serial.println(volts,4);
               delay(10);                                  // wait 10 milisecond before the next loop
                                                          // for the analog-to-digital converter to settle
                                                         // after the last reading:
               total = total+ sensorValue;
               totalV = totalV+volts;
             }
         averageV = totalV / numReadings;              // calculate the average Voltage:      
         if (j==1)
         concentration=concentration + 4;
         else if (j>1) 
         concentration = concentration + 2;
         Serial.print(“ \t Average Voltage(Volts) = ”);    // print the result to the serial monitor                                
         Serial.println(averageV,4);                      // send it to the computer as ASCII digits 
         Serial.print(“ Concentration (mM) = ”);          // print the result to the serial monitor                                
         Serial.println(concentration,3);                  // send it to the computer as ASCII digits 
         x[j] = concentration;
         y[j] = averageV;        
         delay(14000);                                   // delay to change sample concentration
        }
                                                         // Exponetial curve fitting t equation y = A+B*exp(C*x)
                                                         // Estimation of c using a and b.
                                                         // initial variables
        for (int j=0; j<maxcount-1; j++)
        {
         dx[j] = x[j+1]-x[j];
         dy[j] = y[j+1]-y[j];
         cx[j] = (x[j]+x[j+1])/2;
         dq[j] = dy[j]/dx[j];
         }
                                                                                     // pre-estimation of b
        b_int_avg =0;        
        for (int jj=0; jj<maxcount-2; jj++)
        {
         b_initial[jj] = (log(dq[jj+1]/dq[jj]))/(cx[jj+1]-cx[jj]);
         }
        for (int jj=0; jj<maxcount-2; jj++)
        {
         b_int_avg = b_int_avg + b_initial[jj];
        }
        b_int_avg = b_int_avg/(maxcount-2);
  Serial.print(“ b-initial = ”);  
  Serial.println(b_int_avg,4);       
                                                                                     // pre-estimaton of a       
        for (int j=0; j<maxcount-1; j++)
        {
         a_initial[j]= dy[j]/(exp(b_int_avg*x[j+1])-exp(b_int_avg*x[j]));       
        }
        a_avg =0;
        for (int j=0; j<maxcount-1; j++)
        {
          a_avg += a_initial[j]; 
        }
        a_avg = a_avg/(maxcount-1);    
        Serial.print(“ a-initial = ”);  
        Serial.println(a_avg,4);    
                                                                  // estimation of c and stanndard error in c;
         for (int j=0; j<maxcount; j++)
         {
          c_initial[j] = y[j] - a_avg*exp(b_int_avg*x[j]); 
         }
          for (int j=0; j<maxcount; j++)
         {
          c += c_initial[j];       
         }
          c=c/maxcount;
         double scaledY = 0.001;
         c=y[maxcount-1]-scaledY;
         double deltaC =0;
         if (a_avg>0)
             deltaC = -scaledY;                                  //move away from asymptote first
           else 
             deltaC = scaledY;
         double abortt=0;
         abortt = abor(y,c);     
         while (abortt>0)
               {
               c=c+deltaC;
               abortt = abor(y,c);
               }
    Serial.print(“ c-initial = ”);  
    Serial.println(c,4);   
  double Bvar1=0;
  double Bvar2=0;
  Bvar1=Devi(c,y,x,dx);
  deltaC=-deltaC;
  Serial.print(“ delta C = ”);  
  Serial.println(deltaC,4);

  double w = 0;   
while(abs(deltaC)>0.00001 && w<100)
  { w=w+1;
    Serial.print(“ w = ”); 
    Serial.print(w);
    Serial.print(“ deltaC = ”); 
    Serial.println(deltaC,6);
    Serial.print(“ c = ”); 
    Serial.println(c,6);
    Serial.print(“ Bvar1 = ”);  
    Serial.println(Bvar1,4);  
    abortt = abor(y,c+deltaC);
    Serial.print(“ abort = ”);  
    Serial.println(abortt,4);  
    if (abortt>0)  
       {
        deltaC=deltaC/2;
       } 
    else
        {
         c=c+deltaC;
         Bvar2 = Devi(c,y,x,dx);  
         Serial.print(“ Bvar2 = ”);  
         Serial.println(Bvar2,4);
           if (Bvar2<Bvar1)
              {
                Bvar1=Bvar2;
               }
          else   
              {
                deltaC=-deltaC/2; 
               }
        }  
  } 
               // linear regression on ln(y-c) = ln(a) + b*x using estimated c value 
              // consider a linear plot for ln(y-c) vs. x; slope=b and intercept = ln (a) => a = exp (intercept) 
        for (int j=0; j<maxcount; j++)
        {
          lnyc[j] = log(y[j]-c);       
        }
        for (int j=0; j<maxcount; j++)
        {
         xbar+=x[j];
         ybar+=lnyc[j];
         xybar+=x[j]*lnyc[j];
         xsqbar+=x[j]*x[j];
        }
        xbar=xbar/maxcount;
        ybar=ybar/maxcount;
        xybar=xybar/maxcount;
        xsqbar=xsqbar/maxcount;
        slope=(xybar-(xbar*ybar))/(xsqbar-(xbar*xbar));
        intercept=ybar-(slope*xbar);
        double x_dev =0;
        for (int j=0; j<maxcount; j++)
            {
             x_dev += sq(x[j] - xbar);
            }
          double y_cap[maxcount];  
        for (int j=0; j<maxcount; j++)
            {        
             y_cap[j] = slope*x[j] + intercept;
             syx += sq(y[j] - y_cap[j]);
            }
  std_err = sqrt(syx/(maxcount-2) );
  stderr_Slope = std_err / sqrt(x_dev) ; 
  stderr_Intercept = stderr_Slope * sqrt(xsqbar);   
  b = slope;
  b_err = stderr_Slope;
  a = exp(intercept);
  a_err = exp(stderr_Intercept);
  Serial.print(“ Exponential fit to the equation: Y = B*exp(C*X)+ A. \n B = ”);  
  Serial.println(a,4);
  Serial.print(“ C = ”);
  Serial.println(b,4);
  Serial.print(“ A = ”);
  Serial.println(c,6);           
                                                //save the slope and intercept data in EPR0M
  EEPROM.put(eeAddress, a);
  eeAddress += sizeof(a);
  EEPROM.put(eeAddress, a_err);
  eeAddress += sizeof(a_err);      
  EEPROM.put(eeAddress, b);
  eeAddress += sizeof(b);
  EEPROM.put(eeAddress, b_err);
  eeAddress += sizeof(b_err);      
  EEPROM.put(eeAddress, c);
  eeAddress += sizeof(c);
  EEPROM.put(eeAddress, c_err);
  eeAddress += sizeof(c_err);      
  delay (1000);
  exit(0);
  }
		

## Appendix B

From Equation (5), the total absorbance of light at wavelength λ1 is:

(A1) $$A\left(\lambda 1\right)=\varepsilon_{\mathrm{Hb},A1c}\left(\lambda 1\right)\times l\times x_{\mathrm{Hb},A1c}+\varepsilon_{\mathrm{Hb},\mathrm{Non}\;A1c}\left(\lambda 1\right)\times l\times x_{\mathrm{Hb},\mathrm{Non}\;A1c}$$

Similarly, at wavelength λ2,

(A2) $$A\left(\lambda 2\right)=\varepsilon_{\mathrm{Hb},A1c}\left(\lambda 2\right)\times l\times x_{\mathrm{Hb},A1c}+\varepsilon_{\mathrm{Hb},\mathrm{Non}\;A1c}\left(\lambda 2\right)\times l\times x_{\mathrm{Hb},\mathrm{Non}\;A1c}$$

The molar absorption coefficients are depended, but the path length and concentrations are independent of on wavelength (λ). At a fixed concentration (x) and wavelength λ, the change in absorbance with change in path length (l) is given as follows:

(A3) $$\delta A\left(\lambda \right)=\;\delta (\varepsilon_{\mathrm{Hb},A1c}\left(\lambda \right)\times l\times x_{\mathrm{Hb},A1c}+\varepsilon_{\mathrm{Hb},\mathrm{Non}\;A1c}\left(\lambda \right)\times l\times x_{\mathrm{Hb},\mathrm{Non}\;A1c})$$

In humans the pulse pressure leads to the change in the diameter of the arterioles in tissues [34]. Therefore, there is a change in path length leading to a change in the absorbance [35]. The path length change is same for both glycated and non-glycated hemoglobin,

(A4) $$\delta A\left(\lambda \right)=\;(\varepsilon_{\mathrm{Hb},A1c}\left(\lambda \right)\times x_{\mathrm{Hb},A1c}+\varepsilon_{\mathrm{Hb},\mathrm{Non}\;A1c}\left(\lambda \right)x_{\mathrm{Hb},\mathrm{Non}\;A1c})\times \;\delta l$$

The R parameter is given by the ratio of change in absorbance as the path length changes at wavelengths λ1 and λ2. From the equations, it is given by:

(A5) $$R=\;\frac{{\delta A}_{\lambda 1}}{{\delta A}_{\lambda 2}}=\;\frac{(\varepsilon_{\mathrm{Hb},A1c}\left(\lambda 1\right)\times x_{\mathrm{Hb},A1c}+\varepsilon_{\mathrm{Hb},\mathrm{Non}\;A1c}\left(\lambda 1\right)\times x_{\mathrm{Hb},\mathrm{Non}\;A1c})\times \;\delta l\;}{(\varepsilon_{\mathrm{Hb},A1c}\left(\lambda 2\right)\times x_{\mathrm{Hb},A1c}+\varepsilon_{\mathrm{Hb},\mathrm{Non}\;A1c}\left(\lambda 2\right)\times x_{\mathrm{Hb},\mathrm{Non}\;A1c})\times \;\delta l\;}$$

(A6) $$R=\frac{\varepsilon_{\mathrm{Hb},A1c}\left(\lambda 1\right)\times x_{\mathrm{Hb},A1c}+\varepsilon_{\mathrm{Hb},\mathrm{Non}\;A1c}\left(\lambda 1\right)\times x_{\mathrm{Hb},\mathrm{Non}\;A1c}\;}{\varepsilon_{\mathrm{Hb},A1c}\left(\lambda 2\right)\times x_{\mathrm{Hb},A1c}+\varepsilon_{\mathrm{Hb},\mathrm{Non}\;A1c}\left(\lambda 2\right)\times x_{\mathrm{Hb},\mathrm{Non}\;A1c}\;}$$

R parameter estimated from the measurements of transmitted light intensity (I) and incident light intensity (I_0_) is given by:

(A7) $$R=\;\frac{{\delta A}_{\lambda 1}}{{\delta A}_{\lambda 2}}=\;\frac{\delta {\left[\ln\frac{I}{I_{0}}\right]}_{\lambda 1}}{\delta {\left[\ln\frac{I}{I_{0}}\right]}_{\lambda 2}}=\frac{{\left[\ln\frac{I\left(l1\right)}{I_{0}\left(l1\right)}-\ln\frac{I\left(l2\right)}{I_{0}\left(l2\right)}\right]}_{\lambda 1}}{{\left[\ln\frac{I\left(l1\right)}{I_{0}\left(l1\right)}-\ln\frac{I\left(l2\right)}{I_{0}\left(l2\right)}\right]}_{\lambda 2}}=\frac{\ln{\frac{I\left(l1\right)}{I\left(l2\right)}}_{\lambda 1}}{\ln{\frac{I\left(l1\right)}{I\left(l2\right)}}_{\lambda 2}}$$

Here, l1 and l2 are the two path lengths for which the change in absorbance is calculated. In our device, $I_{0}\left(l1\right)=I_{0}\left(l2\right)$ at both wavelengths λ1 and λ2.

Therefore the % HbA1c is given by:

(A8) $$\%\;\mathrm{HbA}1c=\frac{x_{\mathrm{Hb},\;A1c}}{x_{\mathrm{Hb},A1c}+x_{\mathrm{Hb},\;\mathrm{Non}\;A1c}}\times 100$$

The molar concentration of glycated hemoglobin ($x_{\mathrm{Hb},A1c}$) and non-glycated hemoglobin ($x_{\mathrm{Hb},\mathrm{NonA}1c}$) in terms of % HbA1c are given by:

(A9) $$x_{\mathrm{Hb},A1c}=\frac{\%\mathrm{Hb},A1c}{100}\times {\;x}_{\mathrm{Hb}}$$

(A10) $$x_{\mathrm{Hb},\mathrm{Non}\;A1c}=\left(1-\frac{\%\mathrm{Hb},A1c}{100}\right)\times x_{\mathrm{Hb}}$$

By substituting the molar concentration from Equations (A9) and (A10) in Equation (A6), we get:

(A11) $$R=\frac{\varepsilon_{\mathrm{Hb},A1c}\left(\lambda 1\right)\times \frac{\%\mathrm{Hb},A1c}{100}\times {\;x}_{\mathrm{Hb}}+\varepsilon_{\mathrm{Hb},\mathrm{Non}\;A1c}\left(\lambda 1\right)\times \left(1-\frac{\%\mathrm{Hb},A1c}{100}\right)\times x_{\mathrm{Hb}}\;}{\varepsilon_{\mathrm{Hb},A1c}\left(\lambda 2\right)\times \frac{\%\mathrm{Hb},A1c}{100}\times {\;x}_{\mathrm{Hb}}+\varepsilon_{\mathrm{Hb},\mathrm{Non}\;A1c}\left(\lambda 2\right)\times \left(1-\frac{\%\mathrm{Hb},A1c}{100}\right)\times x_{\mathrm{Hb}}\;}$$

x_Hb_ cancels out in numerator and denominator and rearrange the expression:

(A12) $$R=\frac{\varepsilon_{\mathrm{Hb},\mathrm{Non}\;A1c}\left(\lambda 1\right)+(\varepsilon_{\mathrm{Hb},A1c}\left(\lambda 1\right)-\varepsilon_{\mathrm{Hb},\mathrm{Non}\;A1c}\left(\lambda 1\right))\times \frac{\%\mathrm{Hb},A1c}{100}\;}{\varepsilon_{\mathrm{Hb},\mathrm{Non}\;A1c}\left(\lambda 2\right)+(\varepsilon_{\mathrm{Hb},A1c}\left(\lambda 2\right)-\varepsilon_{\mathrm{Hb},\mathrm{Non}\;A1c}\left(\lambda 2\right))\times \frac{\%\mathrm{Hb},A1c}{100}\;}$$

Thus, the percentage of glycated hemoglobin is given by,

(A13) $$\%\;\mathrm{HbA}1c=\;\frac{\varepsilon_{\mathrm{Hb},\mathrm{NonA}1c}\left(\lambda 1\right)-R\times \varepsilon_{\mathrm{Hb},\mathrm{NonA}1c}\left(\lambda 2\right)\;\times 100}{R\times \left(\varepsilon_{\mathrm{Hb},\mathrm{NonA}1c}\left(\lambda 2\right)\;-{\;\varepsilon }_{\mathrm{HbA}1c}\left(\lambda 2\right)\right)-\left(\varepsilon_{\mathrm{HbNonA}1}\left(\lambda 1\right)-\varepsilon_{\mathrm{HbA}1c}\left(\lambda 1\right)\right)}$$

## Appendix C

The total absorbance of glycohemoglobin A1c is due to both glycated and non-hemoglobin as follows:

$$A=A_{\mathrm{Hb},\;A1c}+A_{\mathrm{Hb},\mathrm{Non}\;A1c}$$

$$\varepsilon_{\mathrm{Hb}}\times {l*x}_{\mathrm{Hb}}=\varepsilon_{\mathrm{Hb},A1c}\times l\times x_{\mathrm{Hb},A1c}+\varepsilon_{\mathrm{Hb},\mathrm{Non}\;A1c}\times l\times x_{\mathrm{Hb},\mathrm{Non}\;A1c}$$

(A14) $$\varepsilon_{\mathrm{Hb}}\times x_{\mathrm{Hb}}=\varepsilon_{\mathrm{Hb},A1c}\times x_{\mathrm{Hb},A1c}+\varepsilon_{\mathrm{Hb},\mathrm{Non}\;A1c}\times x_{\mathrm{Hb},\mathrm{Non}\;A1c}$$

Substituting Equations (A9) and (A10) from Appendix B, in Equation (A14), and cancelling x_Hb_ on both sides we get:

$$\varepsilon_{\mathrm{Hb}}=\varepsilon_{\mathrm{Hb},A1c}\times \frac{\%\mathrm{Hb},A1c}{100}+\varepsilon_{\mathrm{Hb},\mathrm{Non}\;A1c}\times \left(1-\frac{\%\mathrm{Hb},\;A1c}{100}\right)$$

(A15) $$\varepsilon_{\mathrm{Hb},A1c}=\frac{100\times {\;\varepsilon }_{\mathrm{Hb}}-{\;\varepsilon }_{\mathrm{Hb},\mathrm{Non}\;A1c}\times \;\left(100-\%\mathrm{Hb},A1c\right)}{\%\mathrm{HbA}1c\;}$$
